# Multi-Acupuncture Point Injections and Their Anatomical Study in Relation to Neck and Shoulder Pain Syndrome (So-Called *Katakori*) in Japan

**DOI:** 10.1371/journal.pone.0129006

**Published:** 2015-06-05

**Authors:** Hayato Terayama, Hajime Yamazaki, Teruhisa Kanazawa, Kaori Suyama, Osamu Tanaka, Makoto Sawada, Miho Ito, Kenji Ito, Tadashi Akamatsu, Ritsuko Masuda, Toshiyasu Suzuki, Kou Sakabe

**Affiliations:** 1 Department of Anatomy, Tokai University School of Medicine, Isehara-shi, Kanagawa, Japan; 2 Department of Anesthesiology, Tokai University School of Medicine, Isehara-shi, Kanagawa, Japan; 3 Department of Plastic and Cosmetic Surgery, Tokai University School of Medicine, Isehara-shi, Kanagawa, Japan; Central South University, CHINA

## Abstract

*Katakori* is a symptom name that is unique to Japan, and refers to myofascial pain syndrome-like clinical signs in the shoulder girdle. Various methods of pain relief for *katakori* have been reported, but in the present study, we examined the clinical effects of multi-acupuncture point injections (MAPI) in the acupuncture points with which we empirically achieved an effect, as well as the anatomical sites affected by liquid medicine. The subjects were idiopathic *katakori* patients (n = 9), and three cadavers for anatomical investigation. BL-10, GB-21, LI-16, SI-14, and BL-38 as the WHO notation were selected as the acupuncture point. Injections of 1 mL of 1% w/v mepivacaine were introduced at the same time into each of these points in the patients. Assessment items were the Pain Relief Score and the therapeutic effect period. Dissections were centered at the puncture sites of cadavers. India ink was similarly injected into each point, and each site that was darkly-stained with India ink was evaluated. *Katakori* pain in the present study was significantly reduced by MAPI. Regardless of the presence or absence of trigger points, pain was significantly reduced in these cases. Dark staining with India ink at each of the points in the anatomical analysis was as follows: BL-10: over the rectus capitis posterior minor muscle and rectus capitis posterior major muscle fascia; GB-21: over the supraspinatus muscle fascia; LI-16: over the supraspinatus muscle fascia; SI-14: over the rhomboid muscle fascia; and BL-38: over the rhomboid muscle fascia. The anatomical study suggested that the drug effect was exerted on the muscles above and below the muscle fascia, as well as the peripheral nerves because the points of action in acupuncture were darkly-stained in the spaces between the muscle and the muscle fascia.

## Introduction

*Katakori* is a symptom name that is often used in Japan. There is no clear definition for *katakori*, but it can be summarized as "subjectively, when there is discomfort, spontaneous pain, and mild pain on motion in the neck, upper scapula region, scapula region, or interscapular region; and objectively, when these muscles tense abnormally when palpated, and there are tender points or stiffness in specific sites" [1]. It is understood that “*Katakori* is a combination of subjective sensation and objective sensation" [1], and demonstrates myofascial pain syndrome-like (MPS-like) symptoms in the shoulder girdle. The number of persons with the subjective symptoms of *katakori* (stiff shoulder) in the Japanese government survey of 2013 has increased with advancing age [2]. *Katakori* is higher for both males and females in the all person with subjective symptoms in Japan, is a great matter of national concern. *Katakori* can be induced as part of a systemic disease, but can also be induced even without organic disease [2]. *Katakori* commonly affects posture/balance and lifestyle, and MPS does as well [3]; it possibly develops because of so-called red flags [4, 5] that are caused by musculoskeletal disease in the neck, thoracodorsal region, and/or the shoulder girdle, as well as by malignant tumors, inflammatory disease, and ischemic heart disease. Patients with *katakori* often visit medical organizations, but also may visit civilian agencies for folk therapies such as massage and chiropractic services. Therefore, it is difficult to grasp the overall picture about this symptom. In turn, MPS is characterized by pain in muscle groups associated with the presence of trigger points (TrPs). These points are small hard nodules within a firm taut band of muscle as assessed by palpation with characteristic pain patterns [6]. The syndrome mainly affects adults, both males and females, in Japan. The most commonly affected regions are the occipital region, neck, shoulder, upper back, interscapular region, and lumbar region [7, 8]. In this manner, the presence or absence of TrPs marks a difference in the context of the definitions of *katakori* and MPS. Also, the active TrPs seen in MPS normally restrict the joint range of motion and produce a local twitch response (LTR) caused by mechanical stimuli as well as related pain in the muscles [9, 10]; often, the TrPs are separate from the pain sites [11]. We also have therapies for *katakori* symptoms which do not meet the definition of MPS and TrPs. The TrPs in MPS are often known to coincide with acupuncture points, and Melzack *et al* reported that 71% of acupuncture points coincided with TrPs [12]. Moreover, we empirically have felt that the point of tenderness was present in the acupuncture points of the relevant area of *katakori* as well as the MPS. We have extensive clinical experience with the injection of the acupuncture points in numerous *katakori* patients. Acupuncture points in MPS, which are thus clinically important, could possibly also play a major role in the onset of *katakori*; therefore, we empirically performed multi-acupuncture point injections (MAPI) at various acupuncture points including BL-10, GB-21, LI-16, SI-14, and BL-38 (the World Health Organization (WHO) classification names [13] are shown alongside each acupuncture point). We have noticed that MAPI might be proper therapy for *katakori* meanwhile various therapies could be available to this date. Acupuncture points in this study are conditioned not to patients’ reactions against mechanical (push) stimulations but in a matter-of–fact style on the basis of the definition of acupuncture points. We also emphasize that it is difficult *katakori* from MPS of the shoulder girdle by definition. A solution in MAPI would be developed into musculatures to a certainty, but has not elucidated anatomically to the present. We postulate that the muscle on the shoulder girdle takes part in *katakori*. The present study examined the clinical results in patients whose chief complaint was *katakori*, in whom organic disease was excluded, and who underwent MAPI. Injections were likewise made into cadavers, and basic anatomical research was performed in order to assist with the clinical interpretation. The approach to acupuncture points is also improved symptoms by only stimulation such as acupuncture, dry needling, or saline injection to acupuncture points [12, 14–19]. However, the region effected in the related muscle-muscle groups is larger for injections of anesthetic than acupuncture stimulation. Therefore, anatomical research was carried out at the same time in the present study.

## Materials and Methods

### Clinical study

**Fig 1:**
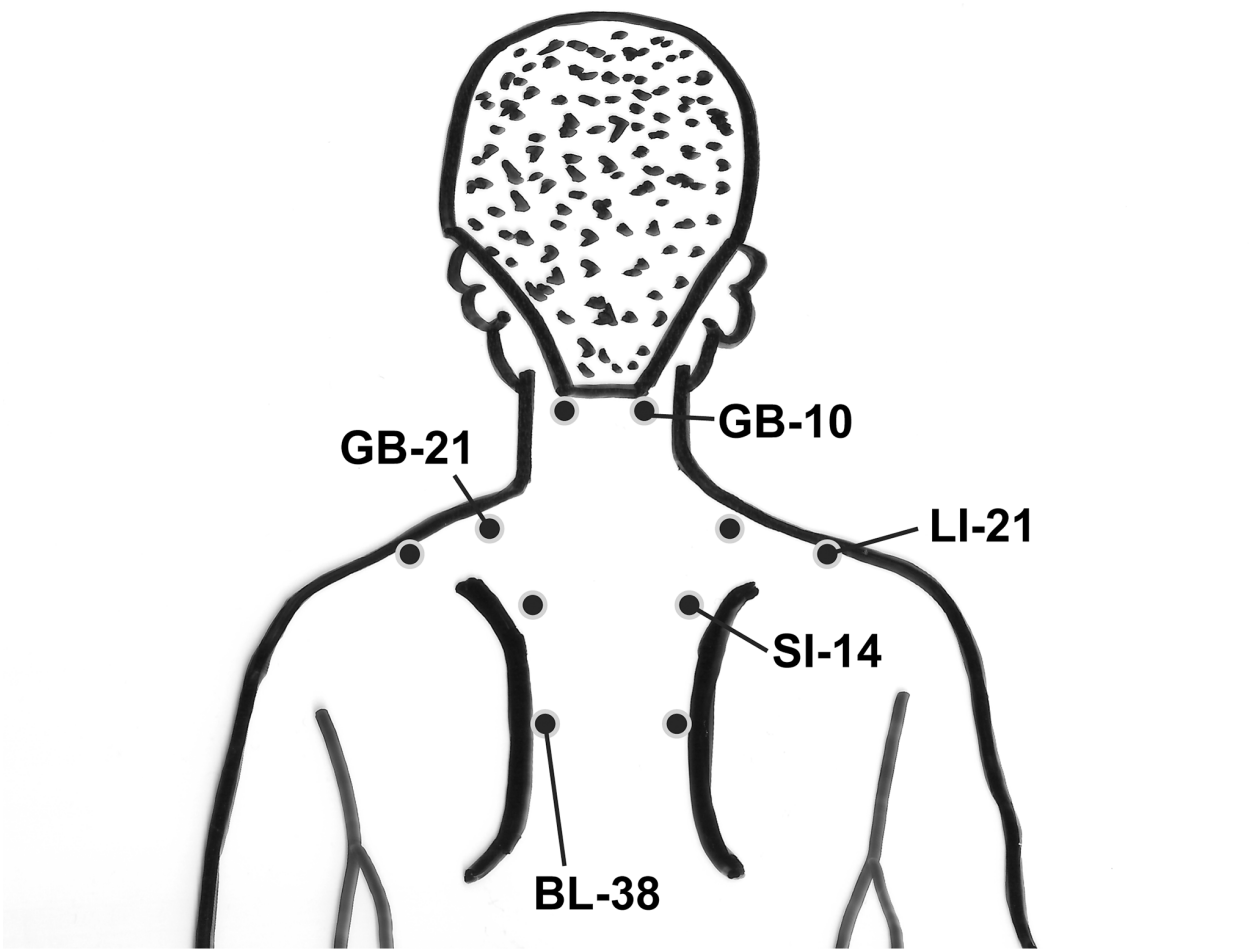
Schema of the injection sites for BL-10, GB-21, LI-16, SI-14, and BL-38. The red circles in the figure show the acupuncture point sites as follows: BL-21, outer margin of the suboccipital trapezius muscle; GB-21, midpoint of the transverse process of the seventh cervical vertebra (C7) and the outer margin of the acromion; LI-16, the outer margin of the supraspinatous fossa; SI-14, medial side of the superior angle of the scapula; and BL-38, medial margin midpoint of the scapula.

After approval from the Institutional Review Board of the Tokai University School of Medicine (IRB Protocol number: 14R014), we retrospectively studied nine *katakori* patients without an underlying disease (male: female = 6:3, mean age = 57.1 ± 2.8 years) from January 4, 2013 to December 28, 2013. Because this study was a retrospective investigation, written/oral informed consent for participation in this study was determined to be unnecessary by the ethics committee. The clinical findings and courses were gathered from past medical records, which limited access to the participants’ backgrounds. All patients were asked about the location of any pain. They underwent general examinations based on their complaints, and were palpated for tender points (PTs) confined to the local area and for the presence or absence of TrPs with related pain. The points used for acupuncture injection were as follows: BL-10, GB-21, LI-16, SI-14, and BL-38 (Fig 1). We decided injection points as only by definition of acupunctures instead of patients responded (tender points or trigger points), so-called ah shi points. A sterile 25-mm-long 25-gauge needle (NN-2525R, Terumo Inc., Tokyo, Japan) was inserted into the acupuncture points (BL-10, GB-21, LI-16, SI-14, and BL-38) at the same time while the patient was seated. The five acupuncture points were selected even in the absence of PTs/TrPs on these acupuncture points. The skin surface area of all patients was prepared with 0.5% w/v chlorhexidine gluconate/ethanol preparation before injection. Injections (5 mL/patient) consisted of mepivacaine hydrochloride injection, 1% w/v (Maruishi Pharmaceutical, Osaka, Japan). All patients were kept quiet and sitting for approximately 15 min after the injection into the acupuncture point. Patients were asked about the presence or absence of adverse events such as mood disquiet during this period, their Pain Relief Score (PRS: change in symptoms after treatment, where 10 is the pain before treatment), and local twitch responses (LTR: When this happens, the TrP treatment worked well, and the larger this response, the closer to a TrP, according to this index). They were also asked about the length of the period of the therapeutic effects from the previous visit when they were revisiting, and information was collected from medical records. Patients who were fearful of injection, uncooperative, or currently taking an anticoagulant drug were excluded from the present study. The change in PRS was analyzed with the Student’s t-test, with the level of significance set to less than 5%. Because there has been no report on the *katakori* treatment that could be referenced, we reluctantly referred to a report on the trigger point injection of MPS in the discussion section.

### Anatomical study

This portion of the study concerned the dissections of three Japanese female cadavers (NO. 1756: 57 years old, uterine cancer; NO. 1758: 78 years old, colon cancer; NO. 1781: 73 years old, gastric cancer) from the anatomy laboratory of the Tokai University School of Medicine (from the Association of Tokai University donations), Japan, during routine educational dissections carried out in 2013 [20–23]. The needles used for acupuncture point injections were 25 gauge. The acupuncture points of the cadaveric neck and shoulder were injected with 1 mL colored solution at a ratio of one part India ink to one part water. The points used for acupuncture injection were BL-10, GB-21, LI-16, SI-14, and BL-38. Needles were injected to a depth of 25 mm, which was deep enough to penetrate the body of the muscle mass. After the injection, the cadaver was maintained in a sitting posture, using crane to lift the upper body by for 15 min (S1 Fig). Gross dissection was performed according to customary procedures. The injected parts were incised, and the acupuncture points of the injected parts were carefully observed.

## Results

### Clinical study

**Fig 2:**
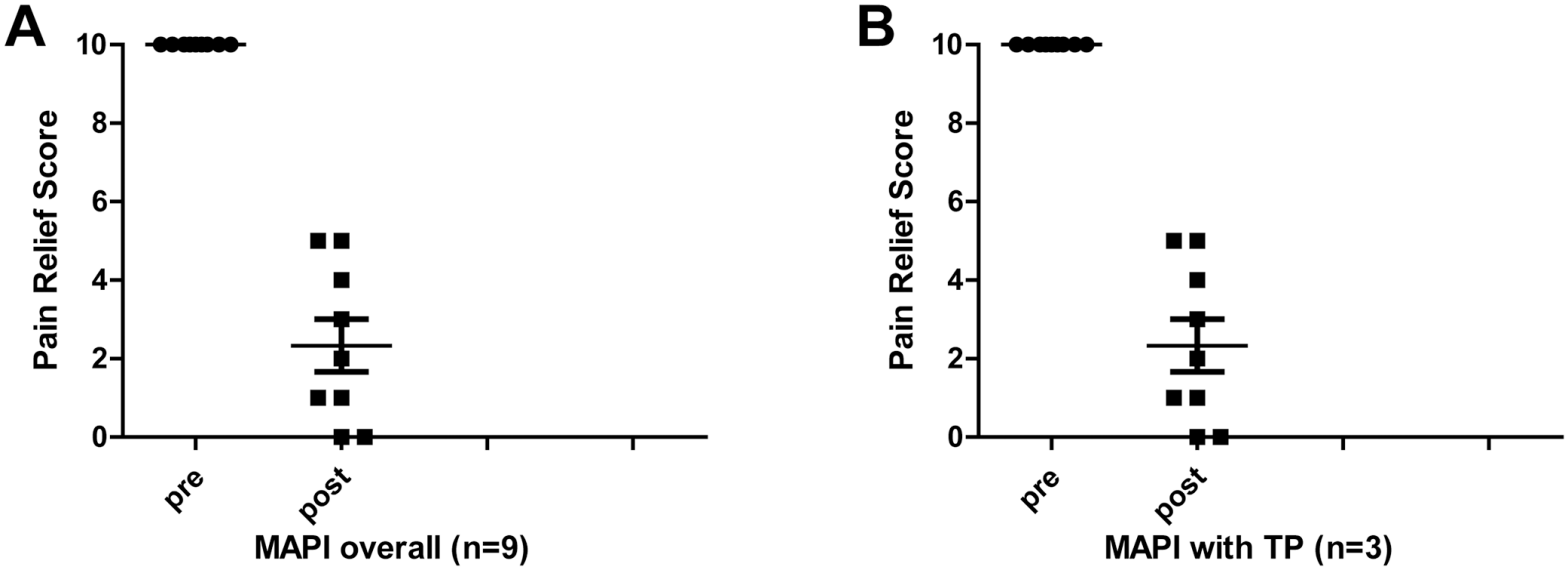
Changes in PRS, when pain intensity before treatment is a 10. A: Overall effect of MAPI (n = 9). After MAPI, the PRS fell to 2.33 ± 2.0 (*p* < 0.0001). B: Effect of MAPI in cases with TrPs (n = 3). After MAPI, the PRS fell to 2.0 ± 2.7 (*p* < 0.05).

Table 1 and Fig 2 summarize PT associated with *katakori* (without related pain) and the presence or absence of TrPs (sites where cords of induration were palpated with pressure stimulus-induced related pain). PTs were present in nine out of nine patients, whereas TrPs were present in three out of nine patients. Similarly, Table 1 also shows the presence or absence of an LTR when MAPI was performed in *katakori* patients, and the change in both the PRS and effect period from this treatment. An LTR did not necessarily appear in the same places as the TrPs, and an LTR was often induced even in the absence of a TrPs. Table 1, which presents the aggregated findings for each acupuncture point, demonstrated an increased tendency for GB-21 and SI-14 to have a PT. The post-MAPI PRS (2.23 ± 2.0) was significantly decreased (*p* = 0.00000296) in the nine patients, and even the three cases in which TrPs were present showed a significant decrease (*p* = 0.0345), with a therapeutic effect obtained regardless of the presence or absence of TrPs. This effect period was 6.33 ± 4.2 days (Fig 2).

**Table 1:** A summary of *katakori* patient backgrounds.

Case	Age	Gender	Tender point area	Area of TrPs	LTR	PRS	Duration (day)
1	56	M	LI16	GB21	GB21	1	10
2	56	M	SI14, GB21	-	-	1	10
3	56	M	BL38, SI14, GB21	-	BL10	5	4
4	63	M	Medial margin of the scapula	-	GB21	0	14
5	59	F	BL38, SI14	-	SI14	4	4
6	56	M	GB21, BL10, SI14, BL38	-	-	3	2
7	57	F	GB21	-	-	2	7
8	53	F	GB21	SI14	GB21	5	3
9	58	M	GB21	BL10	SI14	0	3

A summary of *katakori* patient backgrounds, findings at each acupuncture point (presence or absence of PTs or TrPs), response during MAPI (presence or absence of an LTR), extent of symptom improvement after MAPI (PRS), and duration (days) of symptom improvement after MAPI. The acupuncture names in the table are written based on the WHO notations.

Abbreviations: PT tender point, TrP trigger point, LTR local twitch response, MAPI multi-acupuncture point injection

### Anatomical study

Here we have reported the results from the dissection of the five acupuncture points.

**Fig 3:**
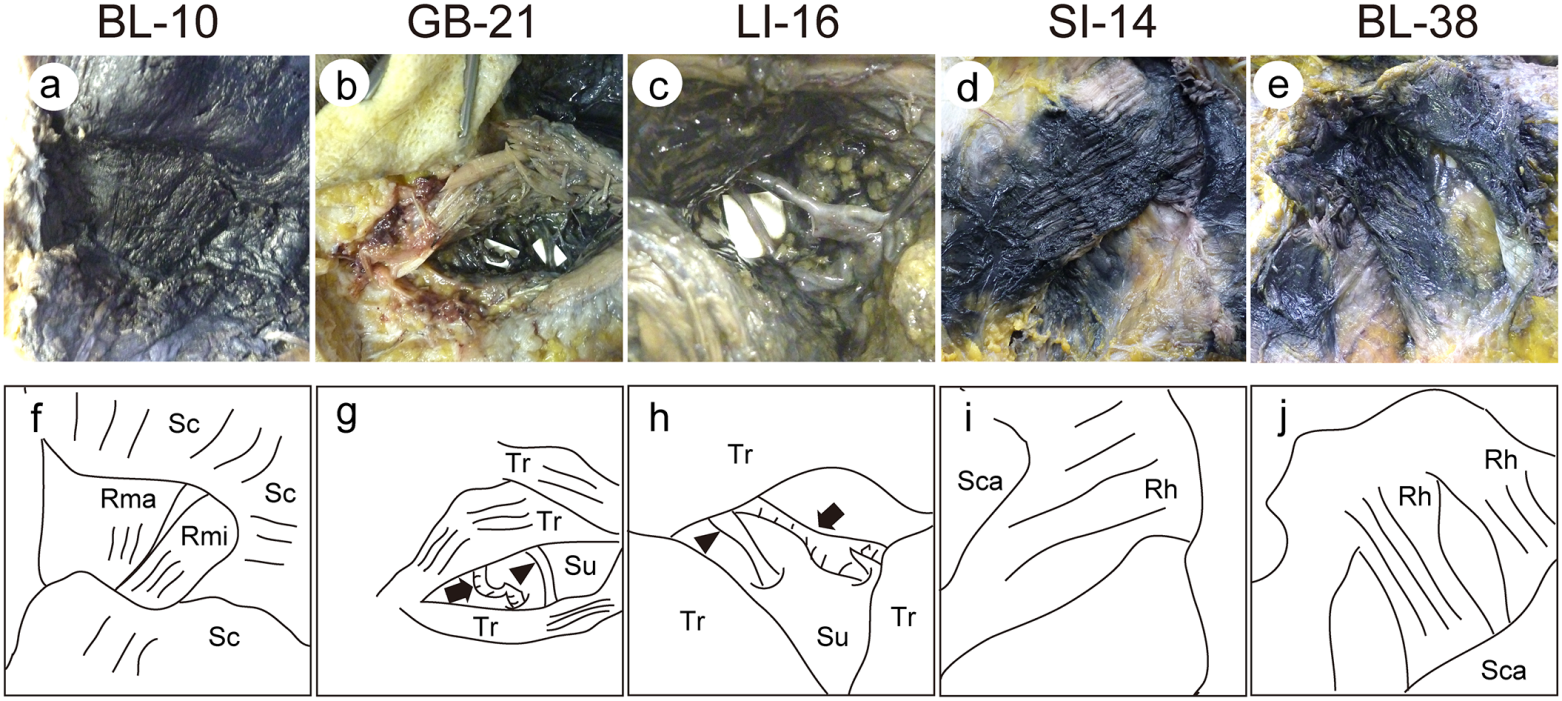
Photographs (a-e) and schematic illustrations (f-j) of the injection sites in BL-10 (a and f), GB-21 (b and g), LI-16 (c and h), SI-14 (d and i), and BL-38 (e and j) in a cadaver. Arrows and arrowheads indicate the suprascapular artery and nerve, respectively. Tr: Fascia of trapezius, Sc: Fascia of semispinalis capitis, Rma: Fascia of rectus capitis posterior major, Rmi: Fascia of rectus capitis posterior minor, Su: Fascia of supraspinatus, Rh: Fascia of rhomboid, Sca: Bone of scapula.

BL-10: The outer margin of the suboccipital trapezius muscle was punctured with a needle [24]. The skin was peeled away, and the greater occipital nerve and lesser occipital nerve were confirmed in the subcutaneous fat layer. After the removal of fat, the trapezius muscle and splenius capitis muscle were reached, but the presence of the India ink could not be confirmed. When the trapezius muscle and splenius capitis muscle were gradually cut, the semispinalis capitis muscle appeared, and the India ink was confirmed. When the semispinalis capitis was gradually cut, the presence of a large amount of India ink was confirmed over the deep rectus capitis posterior major muscle and rectus capitis posterior minor muscle fascia (Fig 3a and 3f).

GB-21: The midpoint of the line on the posterior neck connecting the seventh cervical spinous process and the outer margin of the acromion was punctured with a needle [24]. The skin was peeled away, and the supraclavicular nerve was confirmed in the subcutaneous fat layer. After the removal of fat, the trapezius muscle was reached, but no India ink could be confirmed. When the trapezius muscle was gradually cut, the India ink appeared, and the presence of a large amount of India ink was confirmed over the deep supraspinatus muscle fascia. The suprascapular vessels (arrow) and nerve (arrowhead) were also confirmed (Fig 3b and 3g).

LI-16: The recess on the outside of the supraspinatous fossa between the acromial extremity of the clavicle and the spine of the scapula was punctured with a needle [24]. The skin was peeled away, and the supraclavicular nerve was confirmed in the subcutaneous fat layer. After the removal of fat, the trapezius muscle was reached, but no India ink could be confirmed. When the trapezius muscle was gradually cut, the India ink appeared, and the presence of a large amount of India ink was confirmed over the deep supraspinatus muscle fascia. The suprascapular vessels (arrow) and nerve (arrowhead) were also confirmed (Fig 3c and 3h).

SI-14: The upper back was punctured about 9 cm outside the posterior midline at the same height as the inferior margin of the first thoracic spinous process [24]. The skin was peeled away, and the lateral cutaneous branches and medial cutaneous branches of the intercostal vessels were confirmed in the subcutaneous fat layer, as were the medial cutaneous branches of the intercostal nerves. When the trapezius muscle was reached after the removal of fat, the India ink appeared, and the presence of a large amount of India ink was confirmed over the deep rhomboid muscle fascia (Fig 3d and 3i).

BL-38: The midpoint of the medial margin of the scapula was punctured with a needle [24]. The skin was peeled away, and the lateral cutaneous branches and medial cutaneous branches of the intercostal vessels were confirmed in the subcutaneous fat layer, as were the medial cutaneous branches of the intercostal nerves. When the trapezius muscle was reached after the removal of fat, the India ink appeared, and the presence of a large amount of India ink was confirmed over the deep rhomboid muscle fascia (Fig 3e and 3j). As shown in Table 2, the nerves innervating the muscles where the India ink was observed at BL-10, GB-21, LI-16, SI-14, and BL-38 had anterior branches contributing along with the posterior branches of the cervical nerves.

**Table 2:** The muscles and nerves that are related to acupuncture points.

	Muscle	Nerve	Central nerve
BL-10	semispinalis capitis, rectus capitis posterior major and minor	spinal nerve, suboccipital nerve	posterior ramus: C2-C6, posterior ramus: C1
GB-21	trapezius, supraspinatus	cervical plexus (accessory nerve), suprascapular nerve	anterior ramus: C2-C4, anterior ramus: C5・6
LI-16	trapezius, supraspinatus	cervical plexus (accessory nerve), suprascapular nerve	anterior ramus: C2-C4, anterior ramus: C5・6
SI-14	trapezius, rhomboid	cervical plexus (accessory nerve), dorsal scapular nerve	anterior ramus: C2-C4, anterior ramus: C4-C6
BL-38	trapezius, rhomboid	cervical plexus (accessory nerve), dorsal scapular nerve	anterior ramus: C2-C4, anterior ramus: C4-C6

Summarizes deeply-dyed sites of acupuncture point injection in cadavers.

Deep dyeing was observed in each muscle; nerves innervating the muscles are shown, as are the origin levels of the innervating nerves.

## Discussion

The number of persons who had subjective symptoms of *katakori* in the Japanese government survey of 2013 was 60.2 for men and 125.0 for women per thousand people [2]. In the Japanese government survey of 2013, this symptom was ranked second among men (the first symptom in men was low back pain) and first position in women (second position in women was low back pain); this was almost unchanged as compared to the results of the previous survey from fiscal 2011 [25]. There is a low presence of the subjective symptoms of *katakori* in young people, but that symptom does increase in the middle-aged and elderly populations. In general, the cause of the *katakori* had been a wide range of issues such as musculoskeletal diseases (including the cervical spine), eye diseases, hypertension, and stress. In addition, we have encountered *katakori*'s patients that have had shingles and cancer. The prevalence in a study of migraines of patients that were more than 15 years of age in Japan was estimated to be 8.4% [26]; there has been a case in which *katakori* appeared as a sign of migraine. Tenderness of the skull around the trapezius muscle has also been observed in tension-type headaches [27, 28]. It has been adopted by the latest International Headache Classification, 3β version [29]. Empirically, many patients with tension-type headaches have become aware of the *katakori*. Since an increase in local inflammatory factors were not observed at the tender points in the trapezius muscle with the chronic tension-type headache [30], *katakori* in chronic headache has been identified as factors of the central hypersensitivity [31]. In this way, *katakori* can have a broad range of causes, and to lump all *katakori* together as one disease without consideration for the causes can be dangerous. *Katakori* in the present study is the idiopathic type rather than the symptomatic one. Therefore, the number of cases was limited (retrospective study).

It is not possible to clearly differentiate MPS in the shoulder girdle from *katakori*. However, by definition, the presence of TrPs can be mentioned as one of the things that dictate the difference between the two. The points (NB: ≠TrP, ≠PT) where the participants in the present study who had pressure pain were therefore further divided based on whether or not there was any accompanying related pain, classified as TrPs or (in a narrower sense) PTs, respectively. Of these, TrPs were present in 33.3% (3/9) of the patients. The narrowest sense of PTs, meaning those that did not meet the definition of TrPs, were present in 100.0% (9/9) of the patients, and MPS was included in the subject patients. With MPS, it has been shown that an LTR was induced with the mechanical stimulus of TrP injection [9, 10], but the appearance of an LTR in the present study was similarly observed also in patients where TrPs were not present (nos. 4, 5, and 6). Because an LTR was induced even in PTs where TrPs were not present, an LTR was not a specific reaction for MPS to have TrPs, and most tend to match the acupuncture points (GB-21, BL-10, SI-14). In light of this clinical response, it is possible that neck and shoulder pain syndrome (so-called *katakori*) may be closely related to the broad definition of MPS in the shoulder girdle, and MAPI for idiopathic *katakori* patients may be an effective means of treatment similar to the TrP injections by Graboski *et al* [32], Iwama *et al* [33], Esenyel *et al* [34], and Kiralp *et al* [35]. It is possible that injections that deviate from TrPs exacerbate pain [36], but among the subjects in this study, none had an exacerbation of pain after MAPI. Also, although pain was empirically improved by a TrP injection at one site, there were cases where the next TrP caused pain to develop. As such, the set of five acupuncture points based on our experience—BL-10, GB-21, LI-16, SI-14, and BL-38—may be a replacement for stringent TrP injection, instead of adding a noxious stimulus due to stringent tactile-pressure stimulation while searching for TrPs in MPS patients with pain hypersensitivity. Any difference in effect due to the presence or absence of an LTR cannot be mentioned, but these acupuncture points may be significantly involved in the major pathologies in *katakori* patients, leaving room for investigation based on these points in the future. In addition, whereas the search for TrPs is technically dependent on the level of experience and expertise, treatment by MAPI would also be effective even with physicians with little experience or expertise.

*Katakori* unlike the pathogenesis of MPS, can also be induced by muscle abnormalities. The relationship of *katakori* and MPS may not be clear for the classification of either *katakori* or MPS. In this study, tenderness was present in all *katakori* patients, but tenderness, LTR, and TrPs did not exist together at all acupuncture points. Heine [37] found the relevance of acupuncture points and the fascia corporis superficialis with the neurovascular bundle. In this study, the effect was obtained by injecting all of the selected acupuncture points. Moreover, the effect of MAPI was not only to act at the acupuncture points [14–17, 38] and the neurovascular bundle [37], but also to act directly at the muscle and muscle fascia. Therefore, we also performed anatomical studies with India ink to confirm the muscle distribution of the injected anesthetic. In light of the autopsy findings from the acupuncture points, a drug effect has been found on the trapezius muscle, rhomboid muscle, supraspinatus muscle, semispinalis capitis muscle, the rectus capitis posterior minor muscle, and the rectus capitis posterior major muscle and each of their muscle fascias. Somatic and autonomic nerves are present in the muscles and fascias. As nociceptors of pain, more free nerve endings are particularly distributed in the fascia [39–42]. Therefore, in this study, space of the upper and lower muscle fascia is dyed by India ink. It was suggested that injection into the acupuncture points are affects two muscles involved to *katakori*. Mepivacaine reversibly inhibited the conduction of action potentials in the somatic and autonomic nerves. Moreover, “The effect of inhibition of central sensitivity enhancement” is one of the effects of sensory nerve block. Central nerve related to the each acupuncture points (BL-10, GB-21, LI-16, SI-14, BL-38) has been shown to be involved in the anterior and posterior branches of C1-C6. Effect of MAPI might be trying to suppress the sensitivity of pain by coordinated action of their central nervous system. There also seems to be a contribution to the pain relief of *katakori* due to a pain relief effect and muscle tension relaxation due to nerve blocking as well as an "effect of cutting off the vicious cycle of pain" that may be based on these actions. In light of the autopsy findings, traveling of the suprascapular arteries and nerves, and the vertebral arteries in the suboccipital triangle region was observed in some of the gaps, and therefore careful attention must be paid to acute local anesthetic intoxication and hematoma formation during implementation. To our knowledge, the present study is the first report on issues pertaining to the therapeutic response of MAPI for *katakori* including MPS.

The flexors and extensors are physiologically regulated and controlled by the reciprocal innervation of Ia suppression and Ib suppression. However, it is believed that persistent noxious input to the skeletal muscle causes this control mechanism to fail; it also produces an energy supply shortage in the muscles, where local ischemia develops and causes the release of endogenous pain-producing substances and a decrease in the reaction threshold of polymodal receptors (an energy crisis [43]), leading to a vicious cycle of pain [44]. Breaking this vicious cycle could be the essence of pain treatment. There are many different acupuncture treatments for *katakori* [45–47]. In terms of Eastern medicine, acupuncture treatment is "restoring flow to clogged meridians, which are pathways of vital energy and blood, to improve circulation of vital energy and blood", but in terms of Western medicine, it is believed to have the effect of "improving over-tension and blood flow in the muscles" [45–47]. As such, from this point of view, the acupuncture point injections in the present study may possibly have an additive synergistic effect on pain. The effects of MAPI, however, did not exceed the long-term effects from Esenyel *et al* [34] or Kiralp *et al* [35], who used stretch exercises in combination; the effect period was similar to that of Iwama *et al* (≤7 days) [33] and Graboski *et al* (3.0 ± 3.08 days) [32], who both carried out TrP injection monotherapy. Takakuwa *et al* [48] speculated that circulatory impairment of the trapezius muscle was present in the context of the onset of *katakori*, and they measured the tissue oxygen saturation after exercise with near-infrared spectroscopy in volunteers. They pointed out that the *katakori* group had a significantly greater recovery latency from circulatory impairment associated with an exercise load at the trapezius muscle, especially at a site that corresponded to GB-21, than the non-*katakori* group. The main feature of the integrated trigger point hypothesis is the presence of excess acetylcholine receptors at the neuromuscular junction; acetylcholine stimulates the voltage-gated sodium channels in the sarcoplasmic reticulum and persistently elevates the intracellular Ca^2+^ levels, therefore producing muscle contractures [11, 49]. This occurs as a response not only to direct, acute muscle trauma, but also minor repetitive or chronic muscle trauma [3, 39, 50, 51]. Low-level muscle contraction, which is explained by the Cinderella hypothesis, leads to muscle fiber degeneration, excessive release of Ca^2+^, and inflammatory cytokine release [40, 52, 53], all of which are involved in TrP generation. Persistent muscle contractures that include a TrP damage the local blood vessels, and can even cause hypoxia, decreased pH, and low reflux due to an attenuation of the local oxygen supply; these all contribute to muscle pain and dysfunction [54]. As such, stretching exercises for TrP-related muscle areas, including taut bands, may be beneficial for eliminating shortened sarcomeres [11], which include contraction knots. An increase in intramuscular pressure between low-level muscle contractions is especially likely to occur in muscle attachment sites, and this may lead to hypoxic states and TrP generation [55, 56]. The set of acupuncture points selected in the present study was selected based on the authors' experience, but acupuncture points are rich in the vicinity of muscle attachment sites. Although there may be room to further examine the selected acupuncture points in the future, at this point in time, this method has been shown to produce an immediate effect equivalent to TrP injections in neck and shoulder MPS.

TrPs in MPS have been often known to coincide with acupuncture points, and Melzack *et al* [12] reported that 71% of acupuncture points coincided with TrPs. In the case of MPS of the neck and shoulder, they reported that the trigger points were present in the vicinity of GB-21, TE-15, TE-16, SI-10, and SI-14, respectively. On the other hand, our selected acupuncture points were BL-10, GB-21, LI-16, SI-14, and BL-38, does not coincide with them. Tenderness and the trigger points of *katakori* patients in this study were not present at a high frequency compared to Melzack *et al’*s report [12]. Thus, the pathologies of *katakori* and the neck and shoulder portion of MPS [57] are different. In this study, injections at acupuncture points in patients without tenderness/trigger points had the potential to reduce *katakori*. Each acupuncture point is a non-anatomical pathway that leads to the empirical specific meridians of Chinese traditions. However, the connection of the trigger points has not been demonstrated. For this reason, *katakori* and MPS may need to be considered separately.

The above considerations show that MAPI is effective for the relief of pain and muscle tension at the peripheral level. However, for example, with chronic tension-type headaches, there is no inhibitory mechanism of muscle contraction by the lateral intermediate neurons of the pontine tegmentum, which communicate with the trigeminal motor nuclei of the brain stem and spinal cord [58, 59], with accompanying pain paracranially and in the neck and shoulders. MAPI and TrP injections would presumably be ineffective against *katakori* that was subject to central sensitization in this manner. As such, though symptomatic *katakori* patients, who may have complex conditions, were excluded from the subjects in this study, there may be room for further investigation from this viewpoint in the future.

## Conclusions

MAPI might be effective for idiopathic type *katakori*, to which the myotonia on the shoulder girdle could contribute, from this viewpoint. Idiopathic type *katakori* patients do not have any organic diseases, and might relate to factors of each life background such as posture and stress. This study indicates that muscles in *katakori* patients are innervated by anterior and posterior branch of spinal nerve of C1-C6. These factors concerned in *katakori* onset might overstress to the shoulder girdle muscles. We note that subjects in this study are not symptomatic but idiopathic patients, and these conditions are not equated with. In the future studies would promise to get along with aout anatomical structures in both type of *katakori*.

## Supporting Information

S1 Fig Photograph of the cadaver in a sitting posture by using the crane. We were allowed to wear a belt to the upper body of the cadaver (both armpits), which was lifted by a crane for 15 min.
(ZIP)
