# Supplementary figures and images for: Multi-Acupuncture Point Injections and Their Anatomical Study in Relation to Neck and Shoulder Pain Syndrome (So-Called Katakori) in Japan
Source: PLoS One. 2015 Jun 5;10(6):e0129006. doi: 10.1371/journal.pone.0129006 (PMC4457803; doi:10.1371/journal.pone.0129006)

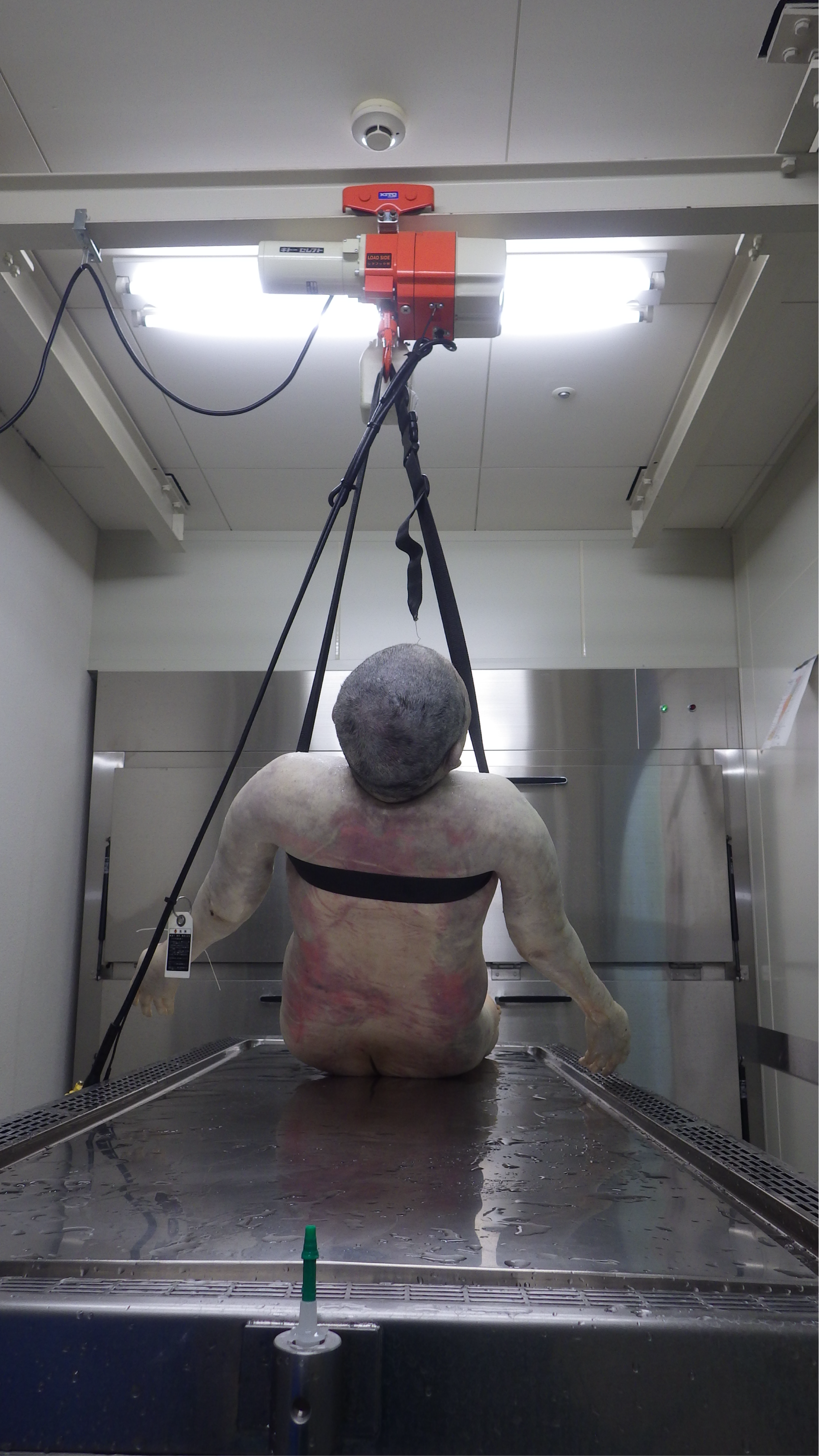

Supplement: S1 Fig — We were allowed to wear a belt to the upper body of the cadaver (both armpits), which was lifted by a crane for 15 min. (ZIP) [file pone.0129006.s001.zip › Figure S1.tif]
